# Insights into thermal sensitivity: Effects of elevated temperature on growth, metabolic rate, and stress responses in Atlantic wolffish (*Anarhichas lupus*)

**DOI:** 10.1111/jfb.16017

**Published:** 2024-12-22

**Authors:** James Hinchcliffe, Jonathan A. C. Roques, Andreas Ekström, Ida Hedén, Kristina Sundell, Henrik Sundh, Erik Sandblom, Björn Thrandur Björnsson, Elisabeth Jönsson

**Affiliations:** ^1^ Department of Biological and Environmental Sciences University of Gothenburg Gothenburg Sweden; ^2^ The Swedish Mariculture Research Center (SWEMARC) University of Gothenburg Gothenburg Sweden; ^3^ Blue Food, Center for Future Seafood University of Gothenburg Gothenburg Sweden

**Keywords:** aerobic scope, Atlantic wolffish, barrier function, metabolism, stress, temperature

## Abstract

The Atlantic wolffish (*Anarhichas lupus*) is a cold‐water fish with potential for aquaculture diversification. To unveil the mechanisms underlying the compromised growth in Atlantic wolffish when reared at higher temperatures, we investigated the relationship between temperature, growth rate, aerobic capacity, stress biomarkers, and gut barrier function. Juveniles acclimated to 10°C were maintained at 10°C (control) or exposed to 15°C for either 24 h (acute exposure) or 50 days (chronic exposure). Fish exposed to 15°C exhibited reduced growth, higher standard, and maximum metabolic rates compared to those at 10°C. In the chronically exposed group at 15°C, metabolic rates were lower than those of acutely exposed fish. The absolute aerobic scope exhibited no significant variation in temperatures; however, the factorial scope showed a notable reduction at 15°C in both acute and chronic exposed groups, aligning with a correlated decrease in individual growth rates. Chronic warming led to increased plasma glucose levels, indicating energy mobilization, but cortisol levels were unaffected. Furthermore, chronic warming resulted in reduced intestinal barrier function, as evidenced by increased ion permeability and a negative potential in the serosa layer. We conclude that warming elevates metabolic rates while reducing intestinal barrier function, thus increasing energy expenditure, collectively, limiting energy available for growth at this temperature from increased allostatic load. Thus, juvenile wolffish maintaining their aerobic scope under thermal stress experience slower growth. This research provides insights for improving the welfare and resilience of wolffish in aquaculture at elevated temperatures and understanding their response to increased environmental temperatures.

## 1 INTRODUCTION

Temperature has been proposed as “the ecological master factor” due to its extensive influence on biochemical and physiological processes in aquatic ectotherms (Brett, 1971). Environmental warming is predicted to drive species ranges poleward; for fishes, this will likely influence abundances and distributions through responses in growth, survival, and reproduction (Hastings et al., 2020; Pörtner & Peck, 2010). As a result, there is growing concern regarding the adaptive capacity of fish to thermal stress, both in natural environments under projected climate change scenarios (Seebacher et al., 2015; Bluemel et al., 2022) and within aquaculture settings (Falconer et al., 2020; Pankhurst & King, 2010).

The concept of aerobic scope (AS) provides valuable insights into an animal's metabolic constraints, influencing its energy allocation to growth and reproduction. Traditionally, AS is defined as the difference between standard and maximum metabolic rates (SMR and MMR, respectively). AS provides a measure of the animal's range of aerobic capacity above its baseline energetic needs and can be expressed as absolute aerobic scope (AAS) or factorial aerobic scope (FAS). AAS is often used for comparing the total metabolic capacity of individuals or species, especially in environments with fixed energy demands. FAS is more useful for comparing metabolic plasticity among species or under different conditions, such as varying environmental stressors (Clark et al., 2013; Gräns et al., 2014; Pörtner & Farrell, 2008).

Wolffish species from the genus *Anarhichas* have been recently proposed as alternative species for cold‐water aquaculture diversification (Falk‐Petersen et al., 1999; Foss et al., 2004; Albertsson et al., 2012; Le François et al., 2002;Le François et al., 2021). In Sweden, efforts have focused on the Atlantic wolffish, *Anarhichas lupus*, which typically has a more southern distribution ranging from northern Europe to Canada and USA. The more northernly distributed relative, the spotted wolffish, *Anarhichas minor*, is found in Norwegian, Icelandic, and North Sea waters at temperatures between −1 and 11°C (Bianucci et al., 2016). Atlantic wolffish are stenothermic and known to change their depth and geographic distribution to stay within their preferred thermal environment (Kulka et al., 2004). Thus, if ocean temperatures continue to rise at current rates, this species will encounter unsuitable thermal conditions at the southern boundaries of its current geographical distribution (Bluemel et al., 2022). Elevated temperature has detrimental effects on both wolffish species (Árnason et al., 2019; Falk‐Petersen et al., 1999; Hansen & Falk‐Petersen, 2002; McCarthy et al., 1998; Moksness, 1994; Pavlov & Moksness, 1997; Rindorf et al., 2020). The optimal temperature (T_opt_, i.e., peak of the thermal performance curve) for growth and survival in the earliest juvenile phase for Atlantic wolffish is reported to be 10.3°C but decreases with age (Falk‐Petersen et al., 1999; Hansen & Falk‐Petersen, 2002). More recently, T_opt_ for growth was established as 12.1°C for juveniles 106–127 days after hatching in the same species (Árnason et al., 2019), and data indicate that serious reduction in growth and health of Atlantic wolffish occurs at 15°C (Árnason et al., 2019; Falk‐Petersen et al., 1999; Hansen & Falk‐Petersen, 2002).

Stress is a common occurrence in both nature and aquaculture and can lead to a reduction in AS due to increased SMR and decreased appetite, limiting energy available for growth (Jobling, 1997). In teleost fish, one of the most important endocrine stress responses is mediated by the hypothalamus‐pituitary‐interrenal (HPI) axis, which results in the production of the glucocorticoid hormone cortisol (the primary stress response; Wendelaar Bonga, 1997). Cortisol has been widely used as an indicator for both acute (Barton, 2006; Fast et al., 2008; Olsen et al., 2008; Roques et al., 2010) and chronic (Le François et al., 2013; Pankhurst et al., 2008; Sundh et al., 2011) stress in fish. However, plasma cortisol levels alone can be difficult to interpret, particularly in chronic situations, as prolonged elevation can lead to habituation or maladaptive responses. It is therefore essential to complement cortisol measurements with secondary stress parameters, such as metabolic markers (glucose, lactate, and osmolality), for a more comprehensive assessment of stress (Wendelaar Bonga, 1997). As part of the secondary stress response, the gut barrier function can be decreased through increased ionic and physical “leakiness” of the gastrointestinal tract (Sundh et al., 2010; Sundh et al., 2018). This can lead to increased gut permeability for macromolecules and bacteria (Knudsen et al., 2008; Sundh et al., 2010), as well as metabolic repercussions due to the increased energy expenditure required to maintain osmotic homeostasis (see Sundell & Sundh, 2012 for a detailed epithelial model of ionic transport in gastrointestinal cells of fish). Spotted wolffish has been suggested to be a slow responder to stress (Lays et al., 2009), with elevated plasma cortisol levels returning to basal levels after 37–168 h following exposure to stressors such as rearing density and air exposure (Le François et al., 2013; Tremblay‐Bourgeois et al., 2010). However, data on stress responses in Atlantic wolffish are lacking.

The present study aimed to investigate the physiological mechanisms underlying the impact of increased temperature on the growth performance of Atlantic wolffish. Specifically, we analysed key performance indicators, including metabolism, endocrine stress response, and gut barrier function to test the hypothesis that reduced growth is caused by constraints on aerobic scope at elevated temperatures, which, in turn, is caused by an increased allostatic load on gastrointestinal function and overall health. Our objectives were to assess the acute (after 24 h) thermal sensitivity of metabolic performance (i.e., SMR, MMR, and AS), as well as the subsequent thermal plasticity following chronic (50 days) warming exposure, and to examine how this relates to stress, health, and growth performance indicators.

## 2 MATERIALS AND METHODS

### 2.1 Experimental animals and holding conditions

Juvenile Atlantic wolffish were obtained from a wild cohort, hatched from fertilized eggs caught during a bottom trawl survey by the Icelandic Marine and Freshwater Research Institute in March 2017. Fish were grown to approximately 7 g and then transported to the experimental facilities at the Department of Biological and Environmental Sciences at the University of Gothenburg, Sweden. Fish were fed daily until visual satiation using a dry marine feed (Skretting amber neptun, grade 1.0 for 7–20 g and grade 2.0 for 20–40 g) until the start of the experiment. Fish were kept in a recirculating aquaculture system (RAS) at 10°C and salinity of 32 ppt under a 12 L:12 D photoperiod. Holding tanks (20 L) had a flow rate of 0.5 L min^−1^ and were fitted with an online temperature monitoring system (Sensdesk, HWgroup, Prague, Czech Republic). All experimental protocols were in accordance with national regulations and covered by ethical permits (165–2015 and 218–2014) approved by the regional ethical committee on animal research in Gothenburg, Sweden.

### 2.2 Experimental design

In February 2018, 112 fish were sedated in water containing tricaine methane‐sulfonate, MS‐222 (Finquel, Scanvacc AS, Norway, 0.16 mg L^−1^), individually tagged intraperitoneally with passive integrated transponder tags (12 mm, Biomark, Boise, USA) and measured for weight and length to the nearest 0.1 g and 0.1 cm, respectively. Fish were then randomly distributed into eight identical experimental round tanks (Ø 29 cm, H 27 cm, filled to 16 L) with 14 fish per tank. The fish density was calculated to be 12.5 kg m^−2^, which is within optimum density ranges of other wolffish species (Le François et al., 2013; Tremblay‐Bourgeois et al., 2010). The initial mean weight and length of the fish were 50.0 ± 8.1 g and 19.0 ± 1.1 cm, respectively. Fish were allowed 14 days to acclimate to the experimental conditions, with temperature maintained at 10°C (10.1 ± 0.2°C). The fish were fed once daily until visual satiation. The remaining pellets were collected 20 min after the feeding session, and daily feed intake (FI) was calculated for each tank. At the start of the experiment, the temperature was increased to 15°C (15.0 ± 0.1°C) in four tanks, whereas four tanks remained at 10°C. The tanks were assigned in duplicates to four experimental treatment groups (*n* = 28 per treatment): an acute, after 24 h exposure to 15°C (15_A_), a chronic (50 days, 15_C_) exposure to 15°C, a control group sampled after 24 h at 10°C (10_A_), and a control group sampled after 50 days at 10°C (10_C_).

### 2.3 Respirometry setup and determination of O_2_ consumption rates

Intermittent‐flow respirometry was used to determine whole‐animal O_2_ consumption rates (*Ṁ*_O2_) as described by Clark et al. (2013). Individual fish were transferred into individual, 1.1‐L acrylic respirometers submerged in a larger experimental tank (1000 L) for respiratory measurements with recirculating aerated seawater at 10.1 ± 0.2°C or 15.0 ± 0.1°C (depending on treatment; *n* = 8 for each treatment group). Fish were fasted for 72 h prior to their introduction into the respirometers. *Ṁ*_O2_ of the undisturbed fish was then measured for 72 h to obtain SMR (see details later). On the third day, the fish were removed from their respective respirometer and subjected to a 10‐min exhaustive chase protocol before being immediately returned to the respirometers. The *Ṁ*_O2_ recordings resumed approximately 1 min after the exhaustive exercise to determine the MMR, as described by Clark et al. (2013).

The partial pressure of O_2_ (P_W_O_2_) in individual respirometers was measured continuously at 1 Hz using a FireSting O_2_ system (PyroScience, Aachen, Germany). The water temperature in the respirometry setup was recorded continuously using a custom‐built temperature logger (EW 7221, CRN Tecnopart, Barcelona, Spain). The recording equipment was connected to a PowerLab 8/30 system (ADInstruments, Castle Hill, Australia), which relayed the output signals to a computer running the data acquisition software LabChart Pro (version 7.2.5, ADInstruments, Castle Hill, Australia). Automated flush pumps refreshed the water in the respirometers for 5 min after each of 15‐min recording periods during which the flush pumps were inactivated. The slope of the decline in the P_W_O_2_ within the respirometers due to fish respiration, between flush cycles, was used to calculate *Ṁ*_O2_ using the following formula:

$${Ṁ}_{\text{O2}}=\left[\left(V_{r}–V_{f}\right)\times {\text{∆P}}_{W}O_{2}\right]/\left(\text{∆t}\times M_{f}\right),$$

where V_r_ is the volume of the respirometer, V_f_ is the volume of the fish (assuming that the overall density of the fish is 1 g per mL of tissue, thus V_f_ = mass of the fish, M_f_), ∆P_W_O_2_ is the change in the air saturation of O_2_ in the water within the respirometer, and ∆t is the time during which ∆ air saturation is measured (Clark et al., 2013). The background microbial respiration was accounted for by subtracting the background P_W_O_2_ slope, measured in each empty respirometer for 24 h after the experimental run. After each experimental protocol, the respirometry equipment was disinfected with 70% alcohol and allowed to dry thoroughly before commencing a new run. This rigorous cleaning procedure was implemented to circumvent the accumulation of bacterial growth in the respirometer prior to the respirometry protocol. The SMR of individual fish was determined as the 20th percentile of *Ṁ*_O2_ measurements recorded during the 72‐h period (Chabot et al., 2016). The MMR was determined as the period with the highest O_2_ consumption rate (i.e., the steepest O_2_‐P_W_O_2_ slope). AAS and FAS for each individual fish was calculated by the following formulas:

AASMMRSMRFASMMR/SMR

The thermal coefficients (Q_10_) for SMR and MMR in acutely and chronically warmed fish were calculated using the equations:

$$Q_{10}={\left[{\mathrm{SMR}}_{2}/{\mathrm{SMR}}_{1}\right]}^{\left[10/\left(15{}^{\circ}C-10{}^{\circ}C\right)\right].}$$

where SMR_1_ is the average SMR at 10°C, and SMR_2_ is the average SMR at 15°C.

$$Q_{10}={\left[{\mathrm{MMR}}_{2}/{\mathrm{MMR}}_{1}\right]}^{\left[10/\left(15{}^{\circ}C-10{}^{\circ}C\right)\right]}$$

where MMR_1_ is the average MMR at 10°C, and MMR_2_ is the average MMR at 15°C.

### 2.4 Growth, biometrics, hematology, and plasma stress physiology

For size measurements, total length (L, cm) and body weight (W, g) were recorded for individual fish. Weight gain (WG, %) and the specific growth rates (SGR) for weight (SGR_W_) and length (SGR_L_) were calculated for the 10_C_ and 15_C_ fish only, using the following formulas:

WG (%) = [(W_F_ – W_I_) × W_I_^−1^] × 100, where W_I_ and W_F_ are the initial (day 1) and final (day 50) weights, respectively. SGR_W_ = 100 × [ln (W_F_ × W_I_^−1^)] × D^−1^ and SGR_L_ = 100 × [ln (L_F_ × L_I_^−1^)] × D^−1^, where W_I_ and L_I_ are the initial (day 1) weights and lengths, respectively, where W_F_ and L_F_ are the final (day 50) weights and lengths, respectively, and D is the number of treatment days (i.e., 50). Condition factor (K) was calculated as K = (W × L^−3^) × 100 at the start (K_I_) and at the end of the experiment (K_F_).

For each tank, FI was recorded daily as g day^−1^, and feed conversion ratio (FCR) was calculated as follows: FCR = FI/TWG, where TWG is the total weight gain per tank in grams.

In parallel to the respirometry recordings, six fish from each holding tank (i.e., *n* = 12 for each treatment group) were sedated with metomidate hydrochloride (Aquacalm, Syndel USA, Ferndale, USA, 10 mg L^−1^) and killed with a sharp blow to the head for blood and tissue sampling. Blood samples were taken from the caudal vessels using a 1‐mL heparinized syringe with a 25‐gauge needle. Haemoglobin concentration (Hb) was determined using a handheld Hb analyser (HemoCue 201+, Ängelholm, Sweden), and values were corrected for fish blood according to Clark et al. (2008). Hematocrit (Hct) was assessed in duplicate by drawing blood into a heparinized capillary tube, which was sealed with critoseal, and then centrifuged for 5 min in a microcentrifuge (Thermoscientific heraeus pico 170, Thermo Fisher Scientific, Waltham, MA, USA). The packed cell volume was read using a Hawksley reader (Lancing, UK) and recorded as percentage of the total blood volume. The mean corpuscular Hb concentration (MCHC, g L^−1^) was calculated as MCHC = [Hb]/Hct × 100.

Remaining blood was immediately centrifuged with a tabletop centrifuge (Thermoscientific hareus pico 17, Thermo Fisher Scientific) for 5 min at 13.5 g, and the plasma was collected and stored at −80°C for later analyses. Plasma glucose and lactate levels (mmol L^−1^) were measured using commercial colorimetric kits (Sigma‐Aldrich, Saint Louis, USA; Instruchemie, Delfzijl, The Netherlands). Plasma osmolality was measured using a cryoscopic osmometer (Advanced Model 3320 Micro‐Osmometer4, Advanced Instruments Inc., Norwood, MA, USA), and plasma cortisol (nmol L^−1^) was assessed using a radioimmunoassay according to Young (1986) with modifications reported in Sundh et al. (2011). The organ masses of the liver (LM), spleen (SM), and heart (HM) were subsequently obtained to calculate hepatosomatic index (HSI), HSI = (LM × BW^−1^) × 100, relative spleen mass (RSM), RSM = (SM × BW^−1^) × 100, and relative ventricular mass (RVM), RVM = (HM × BW^−1^) × 100.

### 2.5 Gill branchial Na^+^/K^+^ ‐ATPase activity

Gill Na^+^/K^+^‐ATPase (NKA) activity was assessed using an NADH‐linked kinetic assay conducted in a 96‐well microplate reader at 25°C for 10 min, following the method outlined in McCormick (1993). This analysis aimed to evaluate any potential alterations in the fish's osmotic capacity. Protein concentration of homogenates was determined using the Pierce BCA Protein Assay (Thermo Scientific, Rockford, IL, USA). Both assays were run on a ThermoMax microplate reader using SoftMax software (Molecular Devices, Menlo Park, CA, USA).

### 2.6 Intestinal barrier function

Intestines were sampled from the 12 fish in the 10_C_ and 15_C_ groups killed for biometric analyses, as described earlier. The body cavity was cut open and the intestine exposed and gently cleaned from mesenteric and adipose tissue. The intestine was then divided in two regions, an anterior mid‐intestine region and a posterior intestine region, according to Hedén (2023). The intestinal segments were gently washed and stored in ice‐cold Ringer solution (160 mmol L^−1^ NaCl, 2.0 mmol L^−1^ KCl, 7.0 mmol L^−1^ NaHCO_3_, 1.6 mmol L^−1^ CaCl_2_, 5 mmol L^−1^ HEPES (4‐[2‐hydroxyethyl]^−1^‐piperazineethanesulfonic acid), 10 mmol L^−1^ d‐glucose, 0.5 mmol L^−1^ l‐lysine, and 20 mmol L^−1^ l‐glutamine, pH [7.8]) and saturated with a gas mixture (99.7% air and 0.3% CO_2_) until further processing. Intestinal barrier and transport functions were assessed in parallel, immediately after sampling, using an in vitro Ussing chamber setup according to Sundell et al. (2003), with modifications as described by Sundell and Sundh (2012). The intestinal tissue was mounted into the Ussing chamber, where they were allowed 60 min of recovery to reach a steady state. The experiment was then started by renewing the Ringer solution on the serosal side and replacing the Ringer solution on the mucosal side with Ringer containing ^14^C‐mannitol (2.14 MBq mol^−1^) (Perkin Elmer; www.perkinelmer.com) and ^3^H‐l‐lysine (1.18 MBq mol^−1^). The sampling was initiated by withdrawing 100 μL samples from the mucosal and serosal half‐chambers. Simultaneously, 100 μL samples were drawn from the serosal half‐chamber, at 20, 25, 30, 60, 80, 85, and 90 min after in exchange for new fresh Ringer. Radioactivity was assessed in a liquid scintillation counter (Wallac 1409, Turku, Finland), after 5 mL of Ultima Gold was added (PerkinElmer Life and Analytical Sciences, Downers Grove, IL, USA). The intestinal barrier function was measured as the apparent paracellular permeability coefficient (P_app_, cm s^−1^) of the hydrophilic marker molecule ^14^C‐mannitol and as the transepithelial resistance (TER, Ω cm^−2^). Together with TER, continuous monitoring of transepithelial potential (TEP, mV) and short‐circuit current (SCC, μA cm^−2^) was done every 5 min and used as control of preparation viability and a measure of active ion transport. P_app_ was calculated as P_app_ = dQ/dt × 1/ACo, where dQ/dt is the appearance rate of mannitol in the serosal compartment of the Ussing chamber (mol s^−1^), A is the area of intestinal surface exposed in the chamber (0.75 cm^2^), and Co is the initial concentration of mannitol on the mucosal side (mol mL^−1^). ^3^H‐lysine transport (mol min^−1^ cm^−2^) was calculated using the appearance rate of labeled lysine on the mucosal chamber side (dQ/dt) and the area of exposure (A).

### 2.7 Statistical analyses

Statistical analysis was performed using SPSS 28 (SPSS, Chicago, USA). All parameters were analysed and compared within the discrete treatments among groups, and survival is expressed as a percentage. Assumptions of normality and homogeneity of variance were assessed using the Shapiro–Wilk test and Levene's test, respectively, followed by individual inspection of data distribution curves. Variables failing these assumptions were log10 transformed and analysed using log10. Mann–Whitney *U* tests were used for variables displaying heterogeneous variance for which transformations were unsuccessful (FI, plasma osmolality, and cortisol). Independent Student's *t*‐tests were used to compare differences in the assessed variables between temperature treatment groups 15_A_ and 10_A_, whereas 15_C_ was compared to parameters for 10_C_. To investigate any potential acclimation within temperature treatments, paired Student's *t‐*tests were used to compare 10_A_ with 10_C_ and 15_A_ with 15_C_. Pearson's correlation analyses were performed to investigate potential relationships between AAS and FAS on SGR in the chronically acclimated groups only. For barrier function measurements, a two‐way ANOVA was carried out to test for the effects of the fixed factors temperature and gut region. Following inspection of time curves, time points 80–100 min (0–40 min after Ringer changes) were deemed as the steady‐state period for the gut during recordings. Therefore, these time points were used during statistical analysis.

## 3 RESULTS

There were no differences in survival among the treatment groups. Only one fish in the 15_C_ treatment died during the course of the experiment. Presented results represent mean values ± SD.

### 3.1 Metabolism

**FIGURE 1:**
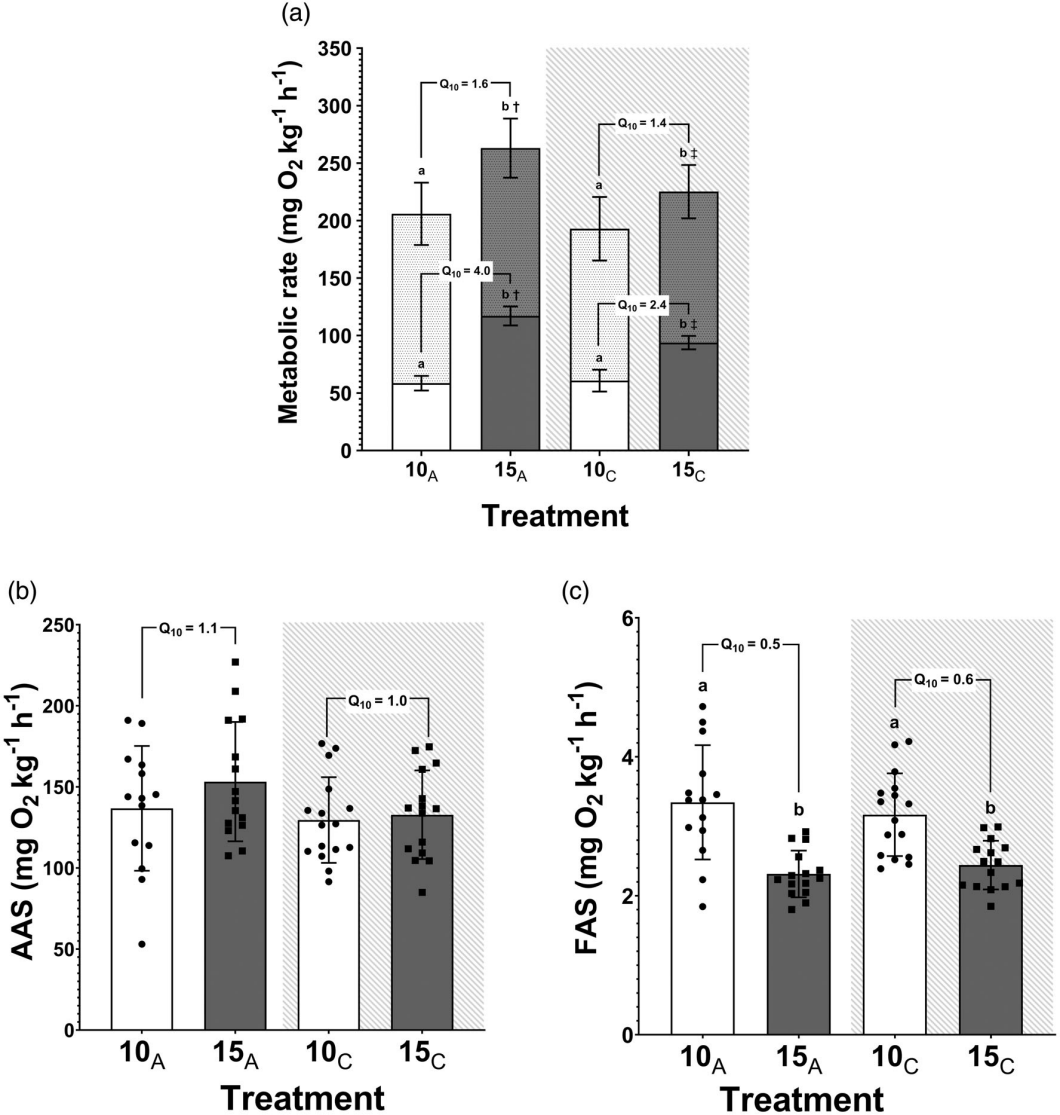
Standard metabolic rate (SMR) and maximum metabolic rate (MMR) (a), absolute aerobic scope (AAS) (b), and factorial aerobic scope (FAS) (c) in the two experimental treatments following acute (10_A_, 15_A_, white background) and chronic exposure (10_C_ and 15_C_, shaded background). All values are displayed as mean ± SD (*n* = 16). Letters (a, b) indicate significant differences between temperature treatments at the corresponding time point, whereas symbols denote time effects specific to each temperature, with # and ## used for time differences at 10°C and † and ‡ for time differences at 15°C.

There was a significant increase in SMR for fish acutely exposed to 15°C relative to fish kept at 10°C (117.1 ± 8.3 vs. 58.5 ± 6.4 mg O_2_ h^−1^ kg^−1^, respectively; *t‐*test, *p* < 0.01), which corresponds to a Q_10_ of 4.0 (Figure 1a). Following chronic warming at 15°C, SMR was still significantly higher relative to fish chronically acclimated to 10°C. However, there was a significant reduction in the thermal sensitivity of SMR when comparing the 15_C_ and 10_C_ treatment groups (93.8 ± 5.8 vs. 60.8 ± 9.5 mg O_2_ h^−1^ kg^−1^, respectively; *t‐*test, *p* < 0.01), which reflected a lower Q_10_ of 2.4 (Figure 1a).

Fish exposed acutely to 15°C had significantly higher MMR relative to fish at 10°C (263.0 ± 25.7 vs. 205.9 ± 27.1 mg O_2_ h^−1^ kg^−1^, *t‐*test, *p* < 0.01; Figure 1a), reflecting a Q_10_ of 1.6. Following chronic warming, MMR was still significantly higher than in fish at 10°C (225.2 ± 23.2 vs. 192.9 ± 27.6 vs. mg O_2_ h^−1^ kg^−1^, *t‐*test, *p* < 0.01), reflecting a Q_10_ of 1.4. Nonetheless, as with SMR, the difference was reduced relative to acutely warmed fish (263.0 ± 25.7 vs. 225.2 ± 23.2 mg O_2_ h^−1^ kg^−1^, respectively; *t‐*test, *p* < 0.01; Figure 1a).

There was no significant effect of temperature on AAS in fish acutely exposed to 10°C or 15°C (136.7 ± 38.5 vs. 153.2 ± 36.8 mg O_2_ h^−1^ kg^−1^, *t*‐test, *p* = 0.25), with a corresponding Q_10_ value of 1.1, and this pattern was similar in the chronically exposed groups (129.5 ± 26.4 vs. 132.8 ± 27.3 mg O_2_ h^−1^ kg^−1^, *t*‐test, *p* = 0.74) and a similar Q_10_ value of 1.0. However, FAS was significantly lower in fish exposed acutely to 15°C relative to 10°C (2.3 ± 0.3 vs. 3.3 ± 0.8, *t*‐test, *p* < 0.01), with a Q_10_ value of 0.5. This observed pattern was also evident in the chronic treatment groups (2.4 ± 0.4 vs. 3.2 ± 0.6, *t*‐test, *p* < 0.01; Figure 1c) and a Q_10_ value of 0.6.

### 3.2 Growth and morphometric indices

Throughout the 50‐day chronic experiment, fish grew from an initial weight of 49.9 ± 10.3 g (10_C_) and 48.6 ± 10.7 g (15_C_) to an average of 71.7 ± 21.0 g (10_C_) or 61.0 ± 14.6 g (15_C_) (Table 1). Although the final weight was not significantly different between the two temperatures (*t‐*test, *p* = 0.22; Table 1), fish maintained at 10°C had a significantly higher SGR for both weight and length (*t*‐test, *p* < 0.01 and *p* < 0.01, respectively), WG (*t*‐test, *p* = 0.02), and lower K_F_ (*t*‐test, *p* < 0.01), compared to fish at 15°C (Table 1). HSI (*t*‐test, *p* = 0.38), RSM (*t*‐test, *p* = 0.32), and RVM (*t*‐test, *p* = 0.30) were not significantly different between temperature treatments after 50 days (Table 1). FI showed a tendency toward a decreased consumption from 5.3 ± 0.2 g day^−1^ (10_C_) to 4.4 ± 0.6 g day^−1^ (15_C_), though not significant (Mann–Whitney *U* test, *p* = 0.09).

**TABLE 1:** Initial weight (W_I_, g), final weight (W_F_, g), weight gain (WG, %), specific growth rate of weight (SGR_W_) and length (SGR_L_), initial condition factor (K_I_), final condition factor (K_F_), feed intake per tank (FI), feed conversion ratio per tank (FCR), and survival per tank (%) of Atlantic wolffish following 50 days of experimental treatments (10_C_ or 15_C_). This is followed by morphometric and physiological characteristics of fish in the four experimental treatments (10_A_, 15_A_, 10_C_, and 15_C_), showing hepatosomatic index (HSI), relative spleen mass (RSM), relative ventricular mass (RVM), and gill Na^+^/K^+^ ATPase (NKA) activity (μmol h^−1^ μg protein^−1^).

Treatment	W_I_	W_F_	WG	SGR_W_	SGR_L_	K_I_	K_F_	FI	FCR	Survival (%)
10_C_	49.9 ± 10.3	71.7 ± 21.0	41.9 ± 22.2^a^	0.7 ± 0.3^a^	0.2 ± 0.1^a^	0.7 ± 0.0	0.7 ± 0.1^a^	5.3 ± 0.2	0.9 ± 0.1	100.0 ± 0.0
15_C_	48.6 ± 10.7	61.0 ± 14.6	25.8 ± 13.4^b^	0.4 ± 0.2^b^	0.1 ± 0.1^b^	0.7 ± 0.1	0.8 ± 0.1^b^	4.4 ± 0.6	1.4 ± 0.1	96.4 ± 3.6

	Treatment	HSI	RSM	RVM	Gill NKA activity
Acute	10_A_	3.7 ± 0.5^#^	0.08 ± 0.01^#^	0.07 ± 0.01	7.5 ± 2.8
	15_A_	3.5 ± 0.5^†^	0.09 ± 0.01^†^	0.07 ± 0.01	7.2 ± 2.0^†^
Chronic	10_C_	3.2 ± 0.3^##^	0.10 ± 0.01^##^	0.08 ± 0.01	7.2 ± 2.8^a^
	15_C_	3.3 ± 0.3^‡^	0.11 ± 0.01^‡^	0.08 ± 0.01	10.9 ± 5.0^‡b^

*Note*: All values are displayed as mean ± SD (*n* = 28 for weights, SGR, K_I_, K_F_, HIS, RSM, RVM, and gill NKA activity; *n* = 2 for FI, FCR, and survival). Letters (a, b) indicate significant differences between temperature treatments at the corresponding time point, whereas symbols denote time effects specific to each temperature, with ^#^ and ^##^ used for time differences at 10°C and ^†^ and ^‡^ for time differences at 15°C.

**FIGURE 2:**
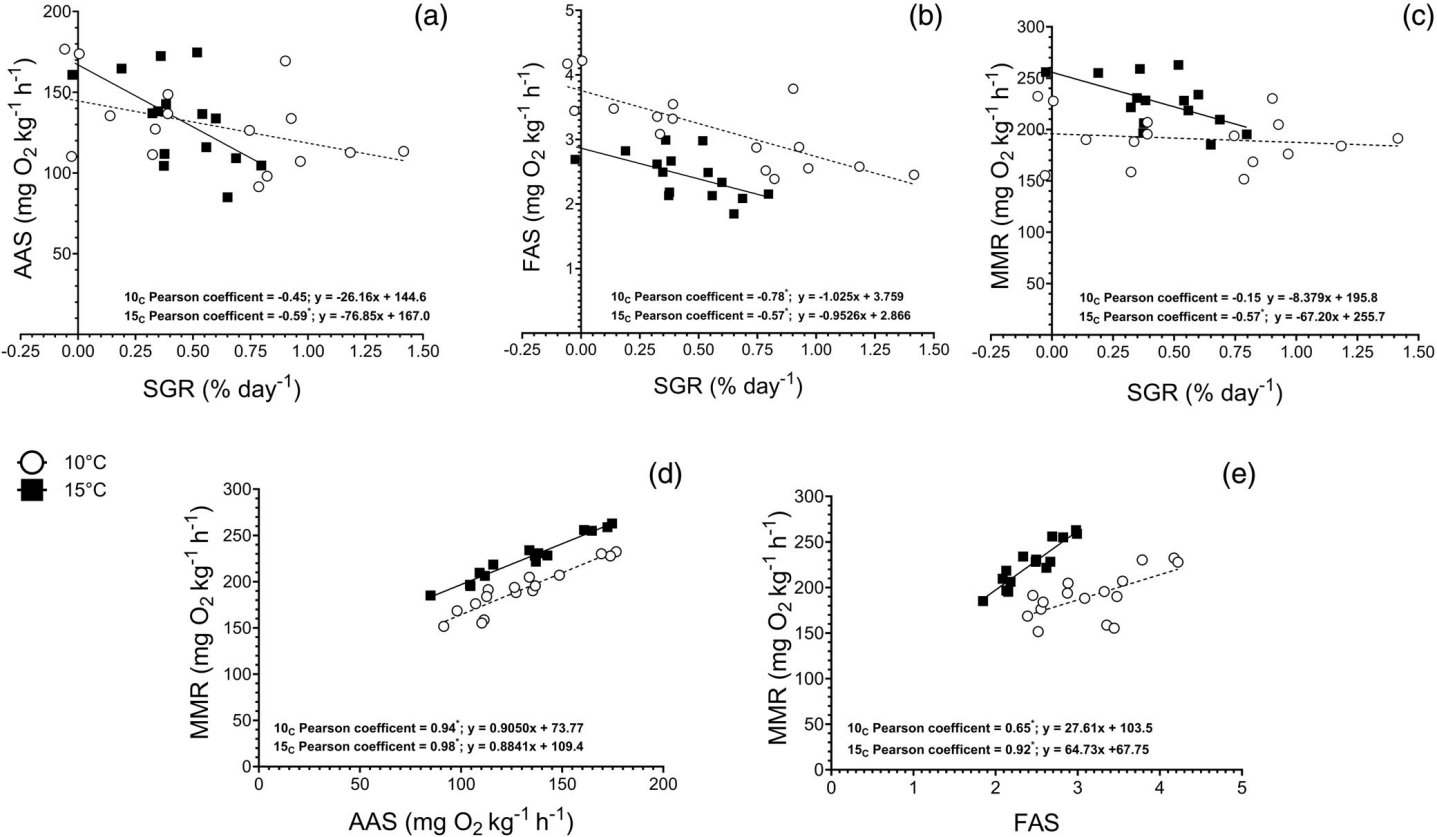
Correlation analysis comparing absolute aerobic scope (AAS) (a), factorial aerobic scope (FAS) (b), and maximum metabolic rate (MMR) (c) with specific growth rate (SGR), as well as correlation analysis comparing MMR against AAS (d) or FAS (e) of individual Atlantic wolffish (*n* = 16), exposed to 10°C (open circles) and 15°C (dark squares). Pearson's coefficient values and linear regression equations for both temperatures are presented below each graph. Asterisks denote significant correlations.

Pearson's correlation analyses revealed that there was a negative correlation between growth rate and both AAS and FAS in both 15_C_ (Pearson's R = −0.59 and −0.57, respectively) and 10_C_ groups (Pearson's R = −0.45 and − 0.78, respectively; Figure 2a,b). For these correlations, fish with higher AAS and FAS showed reduced SGR in fish exposed in 15°C (*p* = 0.02 and *p* = 0.03, respectively) and 10°C for FAS only (*p* < 0.01), though there was also a tendency for AAS (*p* = 0.08). MMR was significantly correlated with SGR in 15_C_ but not 10_C_ (Pearson's R = −0.57, *p* = 0.02 for 15_C_). Both AAS and FAS were grounded on the foundation of MMR, showcasing significant correlations at both 10°C (Pearson's R = 0.94 and 0.65, *p* < 0.01 for AAS and FAS, respectively) and 15°C (Pearson's R = 0.98 and 0.92, *p* < 0.01 for AAS and FAS, respectively; Figure 2d,e).

### 3.3 Plasma health indicators

No significant differences in plasma cortisol levels were found for fish acutely exposed to 10°C and 15°C (17.2 ± 21.6 vs. 14.5 ± 22.0 nmol L^−1^, Mann–Whitney *U*, *p* = 0.60, respectively), as well as for fish chronically exposed to 10°C and 15°C (15.8 ± 21.5 vs. 15.6 ± 21.6 nmol L^−1^, Mann–Whitney *U*, *p* = 0.60, respectively; Table 2). Fish acutely exposed to 10 and 15°C had similar plasma glucose levels (1.0 ± 0.1 vs. 0.9 ± 0.3 nmol L^−1^, *t‐*test, *p* = 0.47; Table 2); however, plasma glucose increased significantly in the chronically exposed groups from 10 to 15°C (0.9 ± 0.2 vs. 1.0 ± 0.1 mmol L^−1^, *p* < 0.01; Table 2).

**TABLE 2:** Haematocrit (Hct, %), haemoglobin (Hb, g L^−1^), mean corpuscular Hb concentration (MCHC, g L^−1^), plasma cortisol (nmol L^−1^), plasma glucose (mmol L^−1^), plasma lactate (mmol L^−1^), and plasma osmolality (mOsmol kg^−1^) of Atlantic wolffish in the four experimental treatments (10_A_, 15_A_, 10_C_, and 15_C_).

	Treatment	Hct	Hb	MCHC	Cortisol	Glucose	Lactate	Osmolality
Acute	10_A_	21.4 ± 0.0	46.3 ± 9.6	2.1 ± 0.4	17.2 ± 21.6	1.0 ± 0.1	1.1 ± 0.3	349.8 ± 11.4
	15_A_	22.3 ± 0.0^†^	46.6 ± 7.6	2.0 ± 0.2	14.5 ± 22.0	0.9 ± 0.3^†^	1.3 ± 0.5	351.1 ± 15.1
Chronic	10_C_	21.3 ± 0.0^a^	42.9 ± 4.5^a^	2.1 ± 0.2	15.8 ± 21.5	0.9 ± 0.2^a^	1.0 ± 0.3	355.5 ± 17.0
	15_C_	26.2 ± 0.0^‡b^	51.5 ± 6.4^b^	2.0 ± 0.2	15.6 ± 21.6	1.0 ± 0.1^‡b^	1.2 ± 0.3	339.0 ± 17.6

*Note*: All values are displayed as mean ± SD (*n* = 12). Letters (a, b) indicate significant differences between temperature treatments at the corresponding time point, whereas symbols denote time effects specific to each temperature, with # and ## used for time differences at 10°C and † and ‡ for time differences at 15°C.

Plasma osmolality and lactate levels were not affected by the temperature or time exposures, (Mann–Whitney *U* test, *p* > 0.05 for osmolality, *t*‐test, *p* > 0.05 for lactate; Table 2). For Hct, a significant increase occurred between 15_A_ and 15_C_ (22.3 ± 0.0 vs. 26.2 ± 0.0%, *t*‐test, *p* = 0.02). Moreover, 15_C_ fish had a significantly higher Hct than the 10_C_ fish (26.2 ± 0.2 vs. 21.3 ± 0.0%, *t*‐test, *p* < 0.05). There was no difference in Hb between 10_A_ and 15_A_ (46.3 ± 9.6 vs. 46.5 ± 7.6 g L^−1^, *t*‐test, *p* = 0.95). But the fish chronically exposed to 15°C showed significantly higher Hb than fish exposed chronically to 10°C (51.5 ± 6.4 vs. 42.9 ± 4.5 g L^−1^, *t*‐test, *p* < 0.05). No effects of exposure time or temperature were found on MCHC.

### 3.4 Gill Na^+^/K^+^‐ATPase activity

Gill NKA activity significantly increased between 15_A_ and 15_C_ (7.2 ± 2.0 vs. 10.9 ± 5.0, μmol h^−1^ μg protein^−1^, *t*‐test, *p* = 0.04). There was also a significant increase in the activity of NKA in fish chronically exposed to 15°C relative to fish exposed to 10°C (7.2 ± 2.8 vs. 10.9 ± 5.0, μmol h^−1^ μg protein^−1^, *t*‐test, *p* = 0.05; Table 1).

### 3.5 Intestinal barrier and transport functions

**FIGURE 3:**
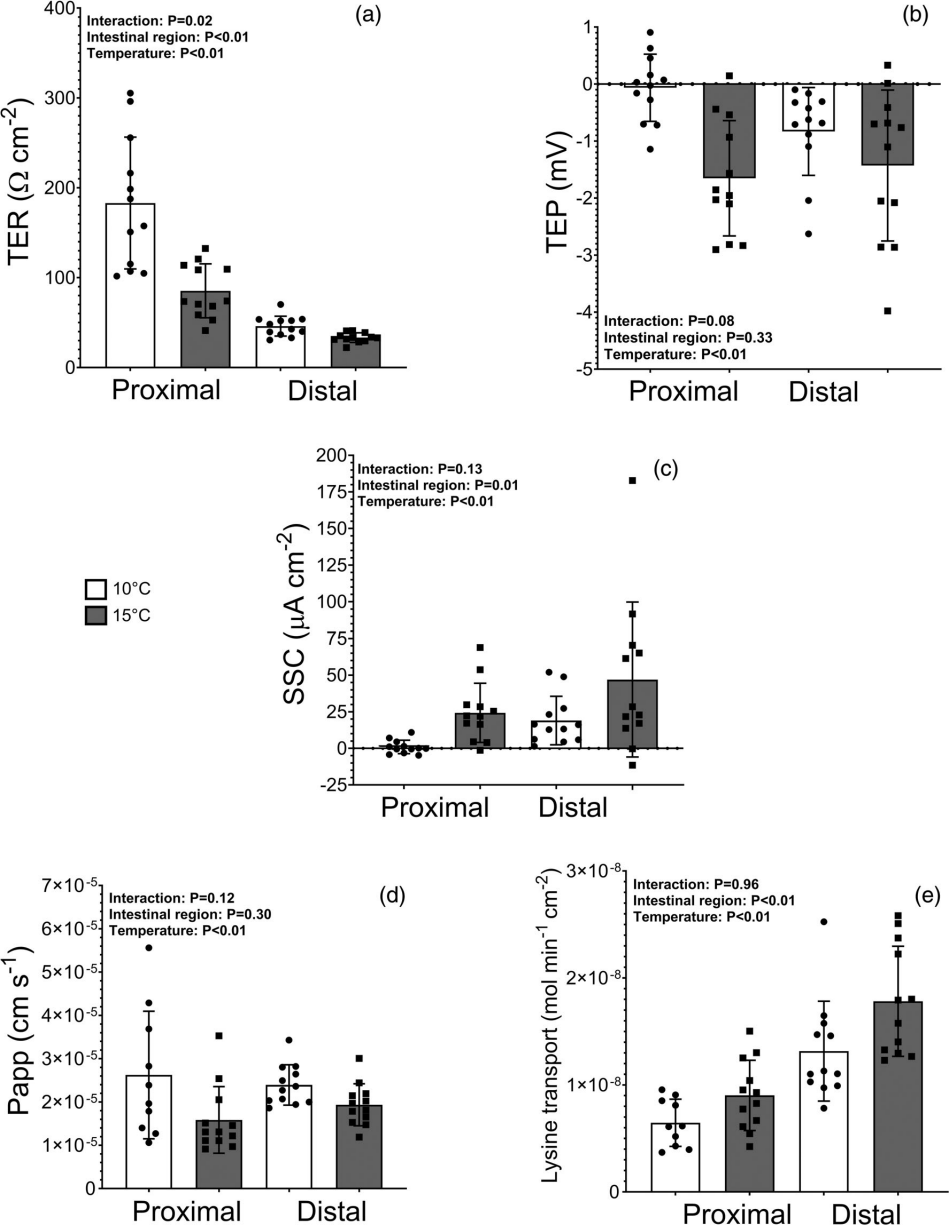
Ussing chambers result in proximal and distal intestine regions of the intestine at 10°C (white) or 15°C (gray) of transepithelial resistance (TER, Ω cm^−2^) (a), transepithelial potential (TEP, mV) (b), short‐circuit current (SCC, μA cm^−2^) (c), mannitol uptake (Papp, cm s^−1^) (d), and lysine uptake (transport, mol min^−1^ cm^−2^) (e). All values are displayed as mean ± SD (*n* = 12). *p*‐Values for main effects and interactions are given in corners of each graph.

There was a significant interaction between gut region and temperature for TER (*p* = 0.02), two‐way ANOVA, followed by main effects for both temperature (*p* < 0.01 and region *p* < 0.01; Figure 3a). TER was significantly reduced at 15°C relative to fish in 10°C (59.3 vs. 114.6 Ω cm^−2^). The two‐way ANOVA also revealed a significantly higher TER in the proximal intestine relative to the distal region in all fish (134.2 ± 15.1 vs. 39.7 ± 2.2 Ω cm^−2^). The interaction showed that TER of the distal regions of the gastrointestinal tract was less affected by an increase in temperature compared to the proximal region. Specifically, when the temperature was raised to 15°C, the TER in the proximal region decreased, whereas the TER in the distal region remained relatively stable.

TEP became significantly more negative in fish chronically exposed to 15°C (−1.5 ± 0.2 vs. 0.4 ± 0.4 mV, two‐way ANOVA, *p* < 0.01; Figure 3b), and SCC significantly increased in fish exposed to 15°C, relative to fish exposed to 10°C (35.6 ± 11.5 vs. 10.0 ± 9.1 μA cm^−2^, two‐way ANOVA, *p* < 0.01; Figure 3c). In contrast, P_app_ showed a significant reduction at 15°C compared to 10°C treatments (1.8 × 10^−5^ ± 0.6 × 10^−5^ cm s^−1^ vs. 2.5 × 10^−5^ ± 0.9 × 10^−5^ cm s^−1^, two‐way ANOVA, *p* < 0.01; Figure 3d). For lysine transport, fish exposed to 15°C also showed a significantly higher uptake compared to fish exposed to 10°C (13.4 × 10^−9^ ± 4.0 × 10^−9^ mol min^−1^ cm^−2^ vs. 9.8 × 10^−9^ ± 3.3 × 10^−9^ mol min^−1^ cm^−2^, two‐way ANOVA, *p* < 0.01; Figure 3e).

There was no significant difference found in TEP between the proximal and distal regions of all fish (−0.9 ± 0.8 vs. −1.1 ± 1.0 mV, two‐way ANOVA, *p* = 0.33; Figure 3b). No significant interaction between temperature and region was found, but a tendency showed that both regions became more negative at 15°C, and more particularly the proximal region (two‐way ANOVA, *p* = 0.08; Figure 3b). Furthermore, SCC was significantly lower in the proximal region compared to the distal region of the intestine in all fish (12.6 ± 11.9 vs. 32.9 ± 33.5 μA cm^−2^, two‐way ANOVA, *p* = 0.01; Figure 3c), and no interaction effect or tendency was found between temperature treatment and gastrointestinal region. No differences in P_app_ were found between the proximal and distal regions of the intestine (2.1 10^−5^ ± 1.1 × 10^−5^ vs. 2.2 × 10^−5^ ± 0.5 × 10^−5^ cm s^−1^, two‐way ANOVA, *p* = 0.29; Figure 3d). For lysine transport, uptake in the proximal regions of the intestine was significantly less than the distal regions (7.7 × 10^−9^ ± 2.6 × 10^−9^ vs. 15.5 × 10^−9^ ± 4.7 × 10^−9^ mol min^−1^ cm^−2^, two‐way ANOVA, *p* < 0.01; Figure 3e), and no interaction was found with temperature treatment.

## 4 DISCUSSION

The current study evaluated the capacity for thermal acclimation of metabolism in juvenile Atlantic wolffish by comparing the effects of acute and chronic thermal exposure. This investigation was conducted considering previous studies on thermal acclimation in other species (Healy & Schulte, 2012; McArley et al., 2017; Pichaud et al., 2017; Sandblom et al., 2014; Sandblom et al., 2016). The main aim was to determine if AS is the primary factor that determines the growth of Atlantic wolffish under thermal stress. Consistent with earlier research (Árnason et al., 2019; Falk‐Petersen et al., 1999; Hansen & Falk‐Petersen, 2002), we demonstrated a reduction in growth at 15°C. However, the current study does not support the hypothesis that reduced AS is the sole determinant of slower growth rates during thermal stress. Instead, our results suggest that other factors are more involved in regulating growth rates under thermal stress, as discussed below.

In the present study, both SMR and MMR significantly increased with acute and chronic increases in temperature, with Q_10_ increasing to 4.0 (SMR) and 1.6 (MMR) between 10_A_ and 15_A_. This response is higher than observations in other fish species, which typically increase *Ṁ*_O2_ with a Q_10_ of 2–3 (Seebacher et al., 2015) with acute warming on SMR. The relatively large Q_10_ increase in the present study indicates that acute temperature elevation from 10 to 15°C represents a severe stress to Atlantic wolffish. In contrast, the Q_10_ for the 15_C_ group was 2.4 and 1.4 for SMR and MMR, which meant a pronounced decrease in the thermal effects on resting metabolism when chronically warmed over 50 days, suggesting a capacity to thermally compensate for the acute effects of warming in Atlantic wolffish. Even so, the Q_10_ of 2.4 for SMR still indicates a relatively limited acclimation to 15°C even after 50 days. Clark et al. (2013) highlighted that metabolic acclimation to warmer or cooler temperatures typically occur within 3 weeks in fish while emphasizing that the physiological mechanisms of thermal acclimation in controlling aerobic scope were poorly understood. Acclimation may take longer time in cold‐water species, as some Antarctic fishes take up to 36 days to acclimate to temperature increases (Peck et al., 2014). The marked effect of warming to 15°C on SMR of Atlantic wolffish was coupled with a tendency (though not significant) of reduced FI at 15°C. At supraoptimal acclimation temperatures, most fish species exhibit a decrease in food consumption, likely due to a loss of appetite (Koskela et al., 1997; Zanuzzo et al., 2020; Jutfelt et al., 2021). Therefore, it is likely that reduced FI also contributed to the reduced growth. The loss of appetite at supraoptimal acclimation temperatures was proposed to be due to an active protection of postprandial residual aerobic scope via reductions in meal sizes (Jutfelt et al., 2021). The regression analysis in the present study suggests that fish exhibiting higher AAS or FAS values showed a discernible reduction in growth rates. This suggests a potential trade‐off or compensatory mechanism, where the energy allocated for maintaining aerobic activity may limit the resources available for growth. Both AAS and FAS were revealed in the present study to be derived from the underlying MMR. Notably, significant correlations were observed at both 10°C and 15°C. Additionally, MMR demonstrated a significant correlation with SGR at 15°C but not at 10°C.

Wolffish are considered to be relatively sedentary (Foss et al., 2004). MMR in sedentary species has been subject to scrutiny due to the chase protocol, which is often utilized to obtain MMR values (Clark et al., 2013; Pörtner, 2014; Jutfelt et al., 2014; Halsey et al., 2018). Therefore, for these species, a realistic MMR may not be obtained by an exhaustive chase exercise and therefore fail to facilitate a maximum metabolic response according to arguments made by Pörtner (2014). The current data have been expressed in absolute terms and as factorial change to allow comparison between the two metrics, as they can run counter to each other and thus yield different conclusions when analysed with the same data. AAS provides an absolute metric, that is, how much O_2_ is available to sustain adenosine triphosphate (ATP) production and aerobic performances, whereas FAS provides a relative scope of how much *Ṁ*_O2_ increases above SMR. Although there were no significant differences in AAS between 10°C and 15°C, a significant reduction was observed when FAS was calculated. For many fish species, FAS often decreases over the inhabited temperature range experienced, whereas AAS displays varying patterns, including bell‐shaped, continuously increasing, or remaining stable (Halsey et al., 2018). In the present study, the similar AAS between groups suggest that, regardless of temperature, the two groups have the same amount of O_2_ available for aerobic activity above SMR. However, the lower FAS of the 15°C fish indicates that the scope to increase aerobic *Ṁ*_O2_ above SMR is constrained. This is supported by the impact on the growth and higher FCR seen at 15_C_. In Atlantic halibut, *Hippoglossus hippoglossus*, growth rate and aerobic scope have distinct and different optimal temperatures (Gräns et al., 2014). This was further substantiated from the current study, where growth rate was reduced, but AAS remained unchanged when fish were acclimated to 15°C. The increased MMR observed in the 15°C treatment could be attributed to the corresponding increase in Hb and Hct, which enhanced the fish's O_2_ transport capacity at this temperature. This suggests that long‐term acclimated Atlantic wolffish may not experience a decline in their O_2_ transport capacity as temperatures approach the upper thermal limit, which, with evidence presented here, we believe is 15°C, consistent with the responses observed in the Atlantic halibut (Gräns et al., 2014).

The current characterization of intestinal barrier function in the Atlantic wolffish shows that barrier function toward ions was significantly reduced in fish exposed to 15°C as observed by a reduction in TER. Impaired barrier function can be induced in fish by several different husbandry‐related practices, including acute handling stress (Olsen et al., 2005, 2008; Sundh et al., 2018), poor water quality (Sundh et al., 2009; Sundh et al., 2010; Sundh et al., 2019), and chronic feed stress (Estensoro et al., 2016; Knudsen et al., 2008; Venkatakrishnan et al., 2019; Vidakovic et al., 2016). The impaired barrier function of wolffish in response to high temperature is in agreement with Sundh et al. (2010), who showed that adverse environmental conditions (low O_2_ levels at low and high temperature), which can occur in sea cages, elicit secondary stress responses in Atlantic salmon, *Salmo salar*, post‐smolts. In that study, intestinal barrier function was significantly impaired by increasing the temperature from 10 to 16°C, even when no primary stress response (plasma cortisol) was observed. In the present study, alongside the decrease in TER, the TEP also showed an elevation in absolute values at 15°C, leading to a more serosa‐negative potential. Transepithelial ion transport is known to be electroneutral (Na^+^ K^+^ 2Cl^−^; Loretz, 1995) and driven by basolateral NKA activity (Sundell & Sundh, 2012). However, the tight junctions are suggested to be more permeable to Na^+^ than Cl^−^, indicating that Na^+^ ions transported across the basolateral membrane can leak back through the tight junctions, resulting in a net increase in Cl^−^ on the serosal side that gives rise to the negative TEP (Loretz, 1995; Sundell & Sundh, 2012). Therefore, the decreased TER and increased serosa‐negative TEP observed at 15°C likely reflect a net accumulation of negative charges (Cl^−^) as a result of an increased leakiness to Na^+^ through tight junctions.

The increase in serosa‐negative mV can be explained by the increase in SCC that occurred at 15°C. SCC represents the overall active transport taking place in the intestinal epithelium, required to bring the TEP to zero. And in this context, it indicates that the actual current generated by the intestine had the same polarity as the negative TEP. However, in the distal intestine of the 15°C fish, despite the higher SCC, the expected further negative shift in TEP was not observed. This was likely due to the decreased barrier function, observed as TER discussed earlier. Where, despite the increased ion transport (via SCC), there was leakage of Na^+^ back into the lumen via the tight junctions, this leakage has potential implications for fluid uptake, as it relies on a strong osmotic gradient created in the lateral intercellular space, which facilitates the movement of fluid from the lumen to the bloodstream (Sundell & Sundh, 2012). The reduced TER and TEP thus suggest that the fluid transport mechanism may be impaired, indicating potential osmoregulatory problems at higher temperatures for Atlantic wolffish. This may be related to overall metabolism, as increased metabolic demands associated with higher active intestinal transport can be required to compensate for intestinal ion loss and maintain necessary ion gradients across the epithelium. Thus, the compromised barrier function and subsequent increased leakage in the distal intestine may play a significant role in shaping the fish's overall metabolic profile. However, as the plasma osmolality was not changed, the increased NKA activity in the 15_C_ group suggests that this intestinal ion leakage was counteracted at the branchial level to preserve homeostasis. The significantly increased intestinal lysine transport of the 15°C fish further indicates more active transport of amino acids at higher temperature. The interplay between active intestinal transport, barrier function, metabolism, and osmoregulation underscores the complexity of physiological responses in fish and highlights the potential cascading effects on their overall energetic demands and homeostasis. In addition to the present study, previous research by Brijs et al. (2018) demonstrated that the gut of rainbow trout (*Oncorhynchus mykiss*) is highly metabolically active. It contributes more than 11% and 24% to the resting aerobic metabolism of the whole animal in a fasted state at 10°C and 15°C, respectively, whereas Morgenroth et al. (2019) showed significant increases in gastrointestinal blood flow occur with acute warming in both freshwater and seawater acclimated trout, further emphasizing the significance of the gut in overall metabolic processes.

In the present study, regional differences in intestinal function were found. The higher TER in the proximal region of the intestine compared to the distal region is in stark contrast with other species, where the posterior intestine of several species (winter flounder, *Pseudopleuronectes americanus*, longjaw mudsucker *Gillichthys mirabilis*, rainbow trout, coho salmon, *Oncorhynchus kisutch*) typically exhibits higher resistance than the anterior intestine (Loretz, 1995; Sundell & Sundh, 2012). The result is surprising, as the proximal intestine is considered the region of active nutrient uptake in teleosts (Loretz, 1995; Sundell & Sundh, 2012). Hellberg and Bjerkås (2000) observed major morphological differences between the anterior and posterior intestine of Atlantic wolffish. More periodic acid–Schiff (PAS)‐positive granules were found in the distal intestine in the apical cytoplasm of epithelial cells, which is consistent with pinocytotic vacuoles believed to be involved in protein absorption (Hellberg & Bjerkås, 2000). Thus, this could indicate that active nutrient absorption occurs in the distal region of the Atlantic wolffish intestine (Hedén, 2023).

Plasma cortisol levels were within the range of reported basal values of below 30 nmol L^−1^ in spotted wolffish (Knutsen et al., 2019; Lays et al., 2009; Le François et al., 2013). The elevation of temperature from 10 to 15°C had no significant effect on cortisol levels, either acutely or chronically. Considering that the sampling was not continuous, it poses a challenge to determine the exact time for a potential cortisol peak that might have occurred in the acute group. For example, in the study by Lays et al. (2009), the authors observed a small increase in cortisol from 27 to 70 nmol L^−1^ after 4–8 h of an acute disturbance. In the case of chronic stress, relying on plasma cortisol alone as a stress measure can be misleading due to potential acclimation to prolonged environmental warming or inhibition of the typical cortisol response from chronic overstimulation of the stress axis (Wendelaar Bonga, 1997). We highlight the necessity to measure this parameter in stress and welfare studies but also advise caution. It is essential to couple it with other secondary parameters, as measuring cortisol alone may not provide a coherent fast and easy method to assess stress and welfare, especially for chronic stress. Similar to plasma cortisol levels, basal plasma glucose levels can vary substantially among teleost fish (Wendelaar Bonga, 1997). For instance, actively swimming species have plasma glucose levels in the range of 3–12 mmol L^−1^ (Boerrigter et al., 2014; Malini et al., 2018), whereas in the present study, glucose levels in all groups, except for 15_C_ fish, were 0.9 mmol L^−1^, which is similar to reports for *Anarhichas minor* (Knutsen et al., 2019; Lays et al., 2009). In the Atlantic wolffish, the mobilization of glucose reached a maximum level of 1.1 mmol L^−1^ after 50 days of chronic exposure to 15°C and suggests a low capacity for energy (glucose) mobilization during stress albeit some was indicated.

## 5 CONCLUSION

This study concludes that reduced AS is not the sole determinant of slower growth rates during thermal stress in Atlantic wolffish. Other factors, such as reduced FI and impaired intestinal barrier function, likely contribute to the reduced growth observed in wolffish at elevated temperatures. The compromised barrier function and increased ion leakage in the distal intestine at 15°C suggest potential osmoregulatory problems and a connection to increased energy demand. The increased NKA activity and stable plasma osmolality suggest that this intestinal ion leakage was counteracted at the branchial level. The study also reveals regional differences in intestinal function, with the distal intestine showing higher resistance and potentially being involved in active nutrient absorption. Basal plasma glucose levels were low in the Atlantic wolffish, indicating a limited capacity for energy mobilization during stress. These findings highlight the complexity of physiological responses in Atlantic wolffish under thermal stress and emphasize the interplay between factors, such as aerobic scope, FI, intestinal barrier function, metabolism, and blood O_2_ transport.

As climate change is leading to widespread rise in ocean temperatures around the globe, the thermal sensitivity of Atlantic wolffish at 15°C that we demonstrate in the present study could be a concern for wild populations, as already indicated recently by Bluemel et al. (2022) and also within aquaculture settings. The observed slow metabolic acclimation capacity from the Q_10_ values 4.0 to 2.4 for SMR after 50 days at chronic warm exposure and impaired growth raises concerns about the ability of Atlantic wolffish to adapt to changing thermal conditions. This could have large consequences for the resilience of wild populations of this sedentary species.

## AUTHOR CONTRIBUTIONS

James Hinchcliffe: conceptualization, data curation, formal analysis, investigation, methodology, software, supervision, validation, visualization, roles/writing—original draft, and writing—review and editing. Jonathan A.C. Roques: conceptualization, data curation, formal analysis, investigation, methodology, software, supervision, validation, visualization, roles/writing—original draft, and writing—review and editing. Andreas Ekström: investigation, methodology, validation, and writing—review and editing. Ida Hedén: investigation, methodology, validation, and writing—review and editing. Kristina Sundell: visualization, funding acquisition, project administration, supervision, and writing—review and editing. Henrik Sundh: roles/writing—original draft, writing—review and editing. Erik Sandblom: conceptualization, methodology, visualization, and writing—review and editing. Björn Thrandur Björnsson: conceptualization, project administration, supervision, visualization, and writing—review and editing. Elisabeth Jönsson: conceptualization, funding acquisition, methodology, project administration, supervision, validation, visualization, roles/writing—original draft, and writing—review and editing.

## FUNDING INFORMATION

This project was part of the NOMACULTURE: “Development of NOvel, high‐quality Marine aquaCULTURE in Sweden—with focus on environmental and economic sustainability” (Dnr DIA 2103‐1961‐27044‐74) funded by MISTRA (The Swedish Foundation for Strategic Environmental Research) and FORMAS (Swedish Research Council For Sustainable Development).

## Data Availability

The data that support the findings of this study are available from the corresponding author upon reasonable request.
